# PRENACEL – a mHealth messaging system to complement antenatal care: a cluster randomized trial

**DOI:** 10.1186/s12978-017-0407-1

**Published:** 2017-11-07

**Authors:** Lívia Oliveira-Ciabati, Carolina Sales Vieira, Ana Carolina Arruda Franzon, Domingos Alves, Fabiani Spessoto Zaratini, Giordana Campos Braga, Jazmin Andrea Cifuentes Sanchez, Lívia Pimenta Bonifácio, Magna Santos Andrade, Mariana Fernandes, Silvana Maria Quintana, Suzi Volpato Fabio, Vicky Nogueira Pileggi, Elisabeth Meloni Vieira, João Paulo Souza

**Affiliations:** 1 Department of Social Medicine, Ribeirao Preto Medical School, University of Sao Paulo, Ribeirao Preto, Brazil; 2 Department of Gynecology and Obstetrics, Ribeirao Preto Medical School, University of Sao Paulo, Ribeirao Preto, Brazil; 3 Women Health Programme, Ribeirao Preto Health Department, Ribeirao Preto, Brazil; 4 Department of Pediatrics, Ribeirao Preto Medical School, University of Sao Paulo, Ribeirao Preto, Brazil

**Keywords:** Antenatal, mHealth, Pregnancy, Syphilis, Adherence, Text messaging

## Abstract

**Background:**

The aim of this study was to determine whether PRENACEL (a bi-directional, mobile-phone based, short text message service (SMS)) increases the coverage of recommended antenatal care (ANC) practices.

**Methods:**

A parallel, cluster-randomized trial in which 20 public primary Health Care Units (PHCUs) were randomly allocated to the intervention (10 PHCUs) or control (10 PHCUs) group. The study population included pregnant women aged 18 or above with a gestational age of 20 weeks or less. Pregnant women receiving ANC in intervention PHCUs were invited through leaflets and posters to register in PRENACEL. Women who registered in PRENACEL received a weekly set of short text messages with health education and health promotion content related to pregnancy and childbirth and were also able to clarify ANC queries through SMS. All women received routine ANC. The primary outcome was the proportion of women with high ANC Score, a composite measure of coverage of recommended ANC practices. Chi-square or Fisher’s exact tests and multivariate log-binomial regression were used to analyze the outcomes.

**Results:**

A total of 1210 eligible women received ANC in the participating PHCUs and took part of this study (770 in the intervention group and 440 in the control group). 20.4% (157/770) of intervention-group women registered in PRENACEL, but only 116 read all messages (73.9% of women who registered in PRENACEL, 116/157). The adjusted intention-to-treat analysis suggested no difference between intervention and control groups in the primary outcome (Adjusted Relative Risk (AdjRR): 1.05 (95% Confidence Interval (CI): 1.00–1.09). Both crude and adjusted per-protocol analysis suggested a positive effect of PRENACEL (Crude RR (95% CI): 1.14 (1.06–1.22), AdjRR (95% CI): 1.12 (1.05–1.21). The multivariate analysis also suggests that the PRENACEL group (women who read all SMS) had higher mean ANC score [48.5 (±4.2) vs 45.2 (±8.7), *p* < 0.01], higher proportion of women with ≥6 ANC visits (96.9% vs. 84.8%, *p* = 0.01), and higher rates of syphilis testing (40.5% vs. 24.8%, *p* = 0.03) and HIV testing (46.6% vs. 25.7%, *p* < 0.01) during ANC.

**Conclusions:**

A bi-directional, mobile-phone based, short text message service is potentially useful to improve the coverage of recommended ANC practices, including syphilis and HIV testing.

**Trial registration:**

Clinical trial registry: RBR-54zf73, U1111–1163-7761.

**Resumo:**

**Introdução:**

O objetivo deste estudo foi determinar se o PRENACEL, um serviço bidirecional de mensagens curtas de texto (SMS) com base na telefonia celular, aumenta a cobertura das práticas recomendadas de cuidados pré-natais (PN).

**Métodos:**

um ensaio paralelo, aleatorizado por conglomerados, no qual 20 unidades básicas de saúde (UBS) foram alocadas aleatoriamente para o grupo de intervenção (10 UBS) ou controle (10 UBS). A população estudada incluiu gestantes com idade igual ou superior a 18 anos com idade gestacional de 20 semanas ou menos. As gestantes que receberam PN em UBS intervenção foram convidadas através de folhetos e cartazes para se inscreverem no PRENACEL. As mulheres que se registraram no PRENACEL receberam um conjunto semanal de SMS com conteúdo de educação e promoção da saúde relacionadas à gravidez e parto e também puderam esclarecer dúvidas relacionadas ao PN através de SMS. Todas as mulheres receberam PN de rotina. O desfecho primário foi a proporção de mulheres com um alto escore de PN, uma medida da cobertura das principais práticas recomendadas no PN.

**Resultados:**

um total de 1.210 mulheres participaram deste estudo (770 no grupo de intervenção e 440 no grupo de controle). 20,4% (157/770) das mulheres do grupo de intervenção demonstraram interesse e foram registradas no PRENACEL, mas apenas 116 leram as mensagens (73,9%, 116/157). A análise ajustada de intenção de tratamento sugeriu ausência de efeito da intervenção no desfecho primário (Risco Relativo (RR) ajustado: 1,05, Intervalo de Confiança (IC) de 95%: 1,00–1,09). A análise por protocolo sugeriu um efeito positivo do PRENACEL [RR bruto (IC 95%): 1,14 (1,06–1,22), RR ajustado (IC 95%): 1,12 (1,05–1,21)]. A análise multivariada sugeriu que as mulheres que leram os SMS apresentaram a maior média do escore de PN [48,5 (±4,2) vs 45,2 (±8,7), *p* < 0,01], maior proporção de mulheres com ≥6 consultas (96,9% vs. 84,8%, *p* = 0,01) e maiores taxas de teste de sífilis (40,5% vs. 24,8%, p = 0,03) e HIV (46,6% vs. 25,7%, p < 0,01) durante o PN.

**Conclusões:**

o sistema PRENACEL é potencialmente útil para melhorar a cobertura das práticas recomendadas de PN, incluindo testes de sífilis e HIV.

**Electronic supplementary material:**

The online version of this article (10.1186/s12978-017-0407-1) contains supplementary material, which is available to authorized users.

## Plain English summary

Providing women with relevant and accurate health information allows them to recognize whether they are receiving adequate antenatal care (ANC), and to engage more effectively with the health system. PRENACEL delivers health promotion and health education content about pregnancy and childbirth through a short text message service (SMS) and allows pregnant women to clarify queries related to ANC. This study was developed to determine whether PRENACEL increases the coverage of recommended antenatal practices. We compared women who received the SMS package in addition to routine ANC to women who received routine ANC alone. Women who received and read the PRENACEL messages were more likely to have increased number of ANC visits and coverage of recommended ANC practices, including syphilis and HIV testing. Our findings suggest that PRENACEL is a potentially useful adjunct to routine ANC, but additional implementation research is needed to expand its reach at the community level.

## Background

On September 2015, 193 United Nations member states adopted the 2030 Agenda for Sustainable Development and set 17 goals to end poverty, protect the planet, and ensure global prosperity. Ending avoidable maternal mortality is one of the targets for ensuring healthy lives and promote well-being, the third Sustainable Development Goal [1]. The government of Brazil has committed to pursue the further reduction of maternal mortality as part of the quest towards achieving the Sustainable Development Goals [2]. It should be noted that despite a remarkable decrease in maternal mortality over the last 25 years, Brazil has not achieved its previous target of reducing maternal mortality to less than 35 maternal deaths per 100,000 live births (the fifth Millennium Development Goal for Brazil) [3]. The determinants of maternal mortality are complex and multifactorial, but quality ANC is key to improve maternal health and reduce morbidity and mortality [4]. Although 98.7% of Brazilian pregnant women have access to antenatal care (ANC), ensuring quality ANC remains a challenge [5–8]. Preventable conditions such as congenital syphilis continue to be important public health issues [6, 7]. According to the “Birth in Brazil” study, 27% of pregnant women had less than six ANC visits, 11.9% were not tested for syphilis, and 18.3% were not tested for HIV [6–8].

Health education, community engagement and healthcare user empowerment can improve quality of care by increasing consumer demand for effective practices, and by allowing users to recognize care deficiencies [9, 10]. However, health education activities can be burdensome for health professionals, and as such are frequently neglected. Mobile health (M-Health) solutions can serve as alternative means of empowering healthcare users, including pregnant women, and encouraging them to become champions for their own health [11, 12].

Previous randomized trials of mobile phone short text message services (SMS) delivering information to pregnant women reported increased numbers of women with four or more ANC visits [13], increased confidence in relation to childbirth, decreased anxiety [14, 15], and decreased perinatal mortality [16]. However, the impact of m-Health interventions in the coverage of evidence-based practices is poorly understood. The objective of this study is to determine whether the use of a bi-directional short message service (PRENACEL) providing information on pregnancy, childbirth, antenatal and intrapartum care and able to answer specific queries of pregnant women increases the coverage of ANC practices recommended by the relevant guidelines in Brazil.

## Methods

### Design and settings

This was a parallel, cluster-randomized trial comparing routine ANC with routine ANC plus PRENACEL. Twenty primary Health Care Units (PHCUs) were randomly allocated either to the intervention or to the control group (ten PHCUs in each group). Participating PHCUs are part of the Brazilian Unified Health System (SUS), which provides free universal health care, including ANC. In addition to the PHCUs, four maternity hospitals providing public health services took part in this study: Hospital das Clínicas de Ribeirao Preto (University Hospital, Ribeirao Preto Medical School, University of Sao Paulo), Mater (Women’s Health Reference Center); Santa Casa de Misericordia de Ribeirao Preto; and the Cidinha Bonini Maternity Hospital (University of Ribeirao Preto - UNAERP). The study methods were reviewed and approved by the relevant administrative authorities and ethics review board of each institution and the Ribeirao Preto city Health Department. The trial is registered at the Brazilian Clinical Trials Registry (REBEC, registration number RBR-54zf73, available at http://ensaiosclinicos.gov.br/rg/RBR-54zf73/).

### Study population

Eligible participants of this study were pregnant women aged 18 or above with a gestational age of 20 weeks or less receiving ANC at selected PHCUs between April and June 2015. We opted to exclude minors due to the additional complexity of obtaining informed consent from minors’ guardians through a phone interview. Women with gestational age above 20 weeks were excluded as the intervention was designed to be implemented as early as possible in pregnancy.

### Study period

This study was implemented from April 2015 and March 2016. Recruitment for the study was conducted in two time periods. In the first recruitment period, between 1st April and 30th June 2015, eligible women were passively recruited from intervention PHCUs. During this time period, pregnant women receiving routine ANC in intervention PHCUs were invited to register in PRENACEL through flyers and posters. Eligible women who voluntarily registered in PRENACEL were invited to participate in the study. In the second recruitment period, all women who gave birth in the participating maternity hospitals between 1st August 2015 and 31st March 2016 had their medical records reviewed for study eligibility. Women who had received ANC at control or intervention PHCUs and that were eligible at the time of the first recruitment period (i.e. pregnant women aged 18 or above with a gestational age of 20 weeks or less receiving ANC at selected PHCUs between April and June 2015.) were invited to participate in the study. Additional file 1: Figure S1 illustrates the study recruitment strategy.

### Intervention

The intervention was carried out at facility level and at individual level. At facility level, the following intervention was implemented at PHCUs allocated to the intervention group: health care personnel participated in a workshop about PRENACEL; posters inviting pregnant women to enrol in PRENACEL were displayed, and health care workers distributed PRENACEL flyers to women attending ANC. At individual level, women who registered at PRENACEL received a weekly set of short text messages via mobile phone and could also send questions and comments related to ANC via short text message to healthcare providers in the research team.

The PRENACEL SMS package was adapted from the Mobile Alliance for Maternal Action (MAMA) antenatal mobile health education program [17]. To adapt the program to the Brazilian context, we identified content requiring translation, adjustment, or exclusion. The selected content was then translated into Brazilian Portuguese and revised according to national and local guidelines [18, 19]. Nine reproductive health specialists reviewed a draft program with revised content. A structured questionnaire was used to collect the specialists’ assessment on the adequacy of proposed messages. Three researchers (ACF, JPS, and EMV) assessed comments and suggestions and prepared a consensus version of the messages. All communication with the panel was carried out individually by e-mail, without identifying other panel members. To assess SMS adequacy, 16 women participated in a pilot program and received four weekly messages for five weeks. We then conducted two focus group interviews to help refine the clarity and usefulness of the messages. All interviews were tape-recorded.

The final SMS package consisted of 148 messages (four per week) sent to pregnant women who registered in PRENACEL. Messages included information about the physiology of pregnancy and childbirth; elements of ANC; postpartum care and contraception; and psychosocial aspects of pregnancy and the postpartum period. Messages were sent according to each woman’s gestational age. After childbirth, no additional messages were sent.

Participant recruitment was accomplished in a passive manner using posters and flyers in intervention PHCUs. Pregnant women interested in registering in PRENACEL had to send a SMS to the PRENACEL message center. The research team contacted the interested woman with a telephone call to the number that had sent the text message, and carried out a computer-assisted interview to assess eligibility. Eligible women were registered in PRENACEL and recruited to the study after providing verbal informed consent. After women agreed to participate, the individual level intervention was initiated. The PRENACEL messages were sent automatically via SISPRENACEL, an information system developed to automatically send SMS according to each woman’s gestational age. Women could also send questions, complaints or feedback via SMS free of charge. Questions were answered by health providers members of the research team. At any time, pregnant women registered in PRENACEL could choose to end their participation and withdrawal their consent to participate in the study. All pregnant women attending intervention PHCUs received routine ANC, regardless of whether or not they registered in PRENACEL.

### Control

In the control PHCUs, pregnant women received routine ANC. They were only approached to participate in the study after childbirth, in the participating maternity hospitals (Additional file 1: Figure S1).

### Randomization

The 20 PHCUs with the highest number of pregnant women receiving ANC in 2013 in Ribeirão Preto city were identified and selected to participate in the study. Cluster randomization was carried out in two stages. The first stage involved identifying two balanced cluster groups composed of 10 PHCUs each, considering the population size of the catchment area and its social vulnerability (estimated based on the number of people receiving Brazilian government income transfer) [20]. Clusters were allocated to each group by drawing lots with Microsoft Excel 2013© software’s randomization function. When the difference between groups (based on population size and social vulnerability) was less than 15%, the two groups were considered balanced. In the second stage, the groups were randomly allocated to be either the intervention or the control group using the Microsoft Excel 2013© randomization function (Microsoft, Redmond, WA, USA). We opted for cluster randomization over individual randomization to minimize contamination bias.

### Outcome measures

In order to assess the impact of the intervention, an ANC score was developed based on practices recommended by the local ANC protocol (Table 1) [18]. The primary outcome was the proportion of women with high ANC score. The ANC score was dichotomized with a cut-off value equal to or greater than 42 points, calculated according to the Brazilian Ministry of Health ANC protocol, from which the local protocol was developed [21]. The ANC score for each woman was generated by reviewing ANC cards during the hospital admission for labour and childbirth. The ANC card is a document given to each pregnant woman, on which health professionals record basic demographic and obstetric characteristics, laboratory results, weight change, uterine fundal height, blood pressure, and other ANC relevant data. Pregnant women are expected to bring the ANC card to each ANC visit and to the maternity hospital for childbirth. Secondary outcomes included the coverage of recommended practices.

**Table 1:** The antenatal care score

Recommended antenatal care practices	Points	Repetition	Total
Antenatal visits	5	6	30
Tetanus immunization	1	1	1
Hepatitis B immunization	1	1	1
Influenza immunization	1	1	1
TDAP immunization	1	1	1
Ferrous sulfate prescription	1	1	1
Folic acid prescription	1	1	1
Dental exam	1	1	1
Educational activities (groups)	1	1	1
Complete blood count	1	1	1
Blood type	1	1	1
Fasting blood glucose	1	1	1
Stool parasitology	1	1	1
Urinalysis	1	2	2
Urine culture	1	2	2
Toxoplasmosis test	1	1	1
Syphilis test	1	3	3
HIV test	1	3	3
Hepatitis B test	1	1	1
OGTT 75 g	1	1	1
Ultrasonography exam	1	1	1
Total			56

TDAP immunization: tetanus, diphtheria, and acellular pertussis; OGTT 75 g: oral glucose tolerance test

### Masking

Participants and health professionals were not masked to the intervention. Participants were free to inform or not inform health professionals of their participation in PRENACEL. Health professionals were therefore not always aware of their patients’ participation in PRENACEL. A statistician who was masked to the study groups conducted data analysis.

### Data collection

Between August 2015 and March 2016, an all-female team of trained data collectors daily visited the maternity hospitals to identify eligible women. After obtaining written informed consent, data collectors used a structured questionnaire to interview eligible women and collect data from medical records, regardless of their PRENACEL registration status. All interviews took place after childbirth and before hospital discharge.

### Data quality

Data was collected using paper forms. Field supervisors conducted a visual inspection of each form seeking missing or inconsistent information before data entry. A data clerk entered data into the Record Electronic Data Capture (REDCap) electronic system [22], which assessed anomalies or inconsistencies in the data. To reduce possible errors, a Java verification script was developed. Data inconsistencies were addressed by re-review of medical records. Inconsistent data that could not be corrected were excluded. Data inconsistencies were found in a total of 0.25% of data points.

### Sample size

The sample size of this study was determined considering that 80% of women receiving routine ANC would achieve a low ANC score. A total sample size of 581 women was required with continuity correction [23] to detect a 15% reduction in the percentage of women with low ANC score as a result of the intervention (α = 5%, power = 80%). Estimating that 25% of women from intervention PCHUs would register to PRENACEL program using passive recruitment approach, 145 women were required for the PRENACEL group, and 436 were required for the control group. Sample calculations were performed using an online calculator [24, 25]. We did not adjust the sample size for the number of clusters (PCHUs), since the analysis was done for each group of PHCUs and not for each individual PHCU.

### Statistical analysis

Data were stratified into three groups: the intervention group (women from intervention PHCUs, regardless of whether or not they had registered to receive PRENACEL SMS); the PRENACEL group (women from intervention PHCUs who registered to receive PRENACEL SMS, received and read the SMS, and did not request discontinuation of service); and the control group (women from control PHCUs). Analysis was conducted both per-protocol (PP), in which women from the PRENACEL group were compared to women from the control group (PRENACEL group versus control group); and intention-to-treat (ITT), in which all women belonging to intervention PHCUs were compared with those from the control PHCUs (intervention PHCUs versus control PHCUs).

Descriptive statistics were used to describe the characteristics of the facilities and individual participants. We assessed categorical variables using the Chi-square or Fisher’s exact tests. The mean ANC score was calculated for each group and compared using the Wilcoxon-Mann-Whitney test. There was an imbalance between groups in terms of social class, marital status, and use of illicit drugs. We therefore controlled for these variables when analyzing the frequency of recommended practices, using log-binomial regression model, performing adjusted per-protocol and intention-to-treat analysis. Although there was no difference between the groups in terms of pregnancy intentions (whether the current pregnancy had been planned or not), this variable was included in the adjusted analysis because of its clinical relevance.

We performed univariate analysis of relative risks (RR) for all possible predictor variables (age, schooling, marital status, drug use, smoking, study group, etc) of high ANC score. Possible predictors with a *p* < 0.10 in univariate analysis were included in the multivariate analysis model (log-binomial regression). These were: study group, social class, marital status, drug use, and pregnancy intentions. Relative risks were presented with a 95% confidence interval (95% CI). The number of women required to receive the PRENACEL SMS package for each additional woman achieving a high ANC score (number needed to treat - NNT) was also determined [26].

The initial phone interview was conducted using Skype software (Microsoft, Redmond, WA, USA). An external statistician blinded to group allocations performed all analyses using SAS software version 9.3 (SAS Institute Inc., Cary, NC, USA). A 5%-significance level was considered for all tests.

## Results

**Fig. 1:**
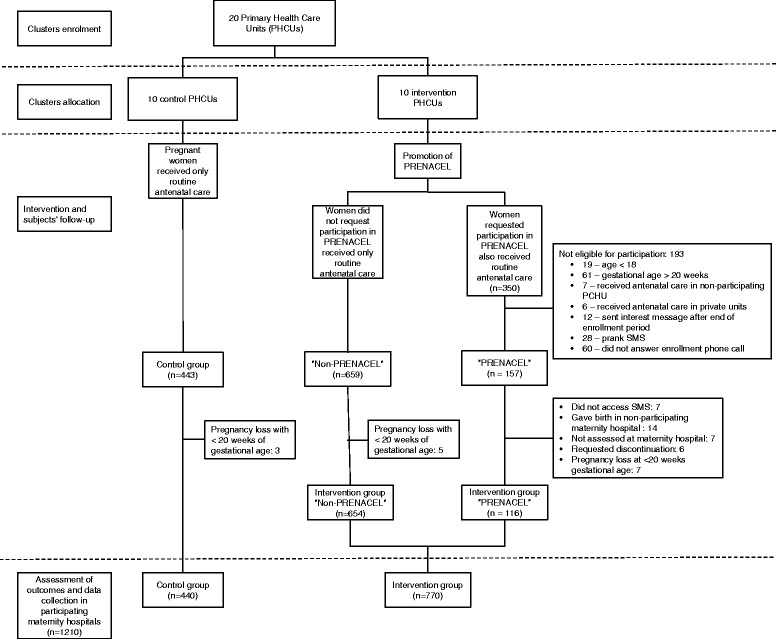
Study Flow diagram

The study flow diagram is presented in Fig. 1. A total of 1210 women received ANC at selected PHCUs and gave birth at participating maternity hospitals (770 women from intervention PHCUs and 440 women from control PHCUs). 20.4% (157/770) of women receiving ANC in intervention group PHCUs registered in PRENACEL, but only 116 of them received and read all messages (73.9% of women registered in PRENACEL, 116/157). Reasons for participant exclusion or loss to follow-up are detailed in the study flow diagram (Fig. 1). There was no difference between clusters in terms of catchment population size and population vulnerability. However, intervention clusters included more PHCUs located in slum areas and fewer PHCUs affiliated with universities compared with control clusters (Table 2).

**Table 2:** Cluster characteristics by study group

Characteristic	Intervention	Control
Catchment population of 2010 [19]	199,564	182,565
Population participating in income transfer program	3842 (1.9%)	3124 (1.7%)
PHCUs located in slum area	8 (80%)	5 (50%)
PHCU affiliated with universities	5 (50%)	9 (90%)
Health care team		
General practitioners and/or Obstetrician-Gynecologists	26 (17.2%)	26 (11%)
Nurses	23 (15.2%)	28 (11.9%)
Community health workers	47 (31.2%)	87 (36.9%)
Nursing Technicians	55 (36.4%)	95 (40.2%)

*PHCUs* Primary Health Care Units

### Intention-to-treat analysis (intervention PCHUs vs. control PCHUs)

Compared to the control group, the intervention group had fewer women from higher social classes (10.1% versus 14.7%, *p* = 0.0002) and fewer women who reported use of illicit drugs (2% vs. 4.3%, *p* = 0.03) (Table 3). Intervention group mean ANC score was higher than the control group [mean ANC (standard deviation (SD)): 46.6 (8.0) vs 45.2 (8.7), p = 0.0002]. The percentage of women attending ≥6 antenatal visits (89.1% vs. 84.8%, *p* = 0.04) was also higher in the intervention group. Women in the intervention group were more likely to receive tetanus-diphtheria-acellular pertussis (TDAP) (39.4% vs 29.8%, *p* = 0.001) and influenza vaccination (35.3% vs. 28%, *p* = 0.01). They were more likely to receive a folic acid prescription (83% vs. 75.9%, *p* = 0.004), to be tested three times for syphilis (34.3% vs. 24.8%, p = 0.001) and for HIV (33.1% vs. 25.7%, *p* = 0.02), and to take a 75 g oral glucose tolerance test (OGTT) (80.3% vs. 72.7%, *p* = 0.003) (Table 4). The adjusted intention-to-treat analysis revealed no statistical difference in the chance of achieving a high ANC score compared to women in the control group (Table 5).

**Table 3:** Sociodemographic characteristics and reproductive history of study participants by group

	Intervention Group		Control Group		
	All IG^a^ (*n* = 770)	PRENACEL^b^ (*n* = 116)	(*n* = 440)	p (ITT)	p (PP)
Age (years)^d^	n (%)	n (%)	n (%)		
18–19	86 (11.5%)	8 (6.9%)	46 (10.6%)	0.76	0.20
20–24	228 (30.4%)	37 (31.9%)	138 (31.9%)		
25–29	220 (29.3%)	44 (37.9%)	125 (28.9%)		
30–34	131 (17.4%)	18 (15.5%)	66 (15.3%)		
≥ 35	86 (11.5%)	9 (7.8%)	57 (13.2%)		
Marital status^d^					
Living with a partner	609 (81.2%)	103 (88.8%)	345 (80%)	0.68	0.04
Not living with a partner	141 (18.8%)	13 (11.2%)	86 (20%)		
Schooling (years)^d^					
< 4	29 (3.9%)	1 (0.9%)	14 (3.3%)	0.30	0.18
5–9	289 (38.9%)	41 (35.3%)	145 (34.1%)		
10–12	384 (51.7%)	63 (54.3%)	244 (57.4%)		
> 12	41 (5.5%)	11 (9.5%)	22 (5.2%)		
Employment^d^					
Paid employment	387 (53.1%)	56 (48.7%)	213 (52.3%)	0.85	0.53
Not employed	342 (46.9%)	59 (51.3%)	194 (47.7%)		
Socioeconomic Class^d^					
Upper/Upper Middle	64 (10.1%)	6 (5.6%)	51 (14.7%)	0.0002	0.02
Middle	359 (56.8%)	70 (65.4%)	222 (64.2%)		
Lower	209 (33.1%)	31 (29%)	73 (21.1%)		
Race^d^					
White	281 (37.4%)	44 (37.9%)	177 (41.3%)	0.66^c^	0.75^c^
Mixed	372 (49.5%)	60 (51.7%)	201 (46.9%)		
Black	88 (11.7%)	11 (9.5%)	46 (10.7%)		
Asian	8 (1.1%)	0 (0%)	3 (0.7%)		
Indigenous	2 (0.3%)	1 (0.9%)	2 (0.5%)		
Previous pregnancies^d^					
0	241 (32.3%)	37 (31.9%)	146 (34.4%)	0.62	0.93
1	244 (32.7%)	37 (31.9%)	124 (29.2%)		
2	118 (15.8%)	19 (16.4%)	66 (15.6%)		
≥ 3	143 (19.2%)	23 (19.8%)	88 (20.8%)		
Previous abortions^d^					
0	619 (83%)	94 (81%)	345 (81.4%)	0.54	1
≥ 1	127 (17%)	22 (19%)	79 (18.6%)		
Pregnancy intentions^d^					
Planned pregnancy	302 (43.1%)	58 (51.8%)	160 (41.7%)	0.68	0.07
Unplanned pregnancy	398 (56.9%)	54 (48.2%)	224 (58.3%)		
Pregnancy risk category^d^					
Low risk, no hospitalization	475 (67.6%)	77 (67%)	279 (71.5%)	0.20	0.41
High risk or hospitalization	228 (32.4%)	38 (33%)	111 (28.5%)		
Behavioural risk factors^d^					
Smoking	89 (12.6%)	12 (10.3%)	59 (15.1%)	0.28	0.25
Alcohol use	140 (19.8%)	14 (12.1%)	66 (16.9%)	0.27	0.27
Illicit drug use	14 (2%)	0 (0%)	17 (4.3%)	0.03^c^	0.02^c^

^a^All IG (Intervention Group) includes all eligible pregnant women who received ANC in intervention primary health care units, regardless of the PRENACEL registration status;

^b^PRENACEL group only includes pregnant women who received and accessed PRENACEL SMS

^c^Values compared using Fisher’s exact test and Chi-Square test

^d^Available sample for this analysis

**Table 4:** Coverage of recommended antenatal care practices

	Intervention Group		Control Group		
	Total^a^ (*n* = 770)	PRENACEL^b^ (*n* = 116)	(*n* = 440)	p (ITT)	p (PP)
Mean ANCS ^c^	46.6 (8.0)	48.5 (4.2)	45.2 (8.7)	0.0002*	<0.0001*
≥ 6 antenatal visits	686 (89.1%)	112 (96.6%)	373 (84.8%)	0.06*	0.01*
Tetanus immunization	272 (35.3%)	39 (33.6%)	145 (33%)	0.44	0.98
Hepatitis B immunization	304 (39.5%)	44 (37.9%)	156 (35.5%)	0.18	0.70
Influenza immunization	272 (35.3%)	36 (31%)	123 (28%)	0.01	0.59
TDAP immunization	303 (39.4%)	45 (38.8%)	131 (29.8%)	0.001	0.08
Ferrous sulfate prescription	629 (81.7%)	104 (89.7%)	352 (80%)	0.52	0.8
Folic acid prescription	639 (83%)	108 (93.1%)	334 (75.9%)	0.03*	0.08*
Dental exam	322 (41.8%)	67 (57.8%)	178 (40.5%)	0.69	0.02*
Educational activities	90 (11.7%)	14 (12.1%)	58 (13.2%)	0.50	0.87
Complete blood count	736 (95.6%)	111 (95.7%)	412 (93.6%)	0.18	0.54
Blood type	716 (93%)	110 (94.8%)	397 (90.2%)	0.11	0.17
Fasting blood glucose	728 (94.5%)	110 (94.8%)	410 (93.2%)	0.40	0.67
Stool parasitology	522 (67.8%)	76 (65.5%)	249 (56.6%)	0.0001	0.10
Urinalysis					
0	52 (6.8%)	4 (3.4%)	40 (9.1%)	0.30	0.13
1	269 (34.9%)	42 (36.2%)	144 (32.7%)		
2	449 (58.3%)	70 (60.3%)	256 (58.2%)		
Urine culture					
0	90 (11.7%)	13 (11.2%)	66 (15%)	0.22	0.58
1	224 (29.1%)	35 (30.2%)	129 (29.3%)		
2	456 (59.2%)	68 (58.6%)	245 (55.7%)		
Toxoplasmosis test	743 (96.5%)	113 (97.4%)	419 (95.2%)	0.35	0.44
Syphilis test					
≤ 2	506 (65.7%)	69 (59.5%)	331 (75.2%)	0.01*	0.03*
3	264 (34.3%)	47 (40.5%)	109 (24.8%)		
HIV test					
≤ 2	515 (66.9%)	62 (53.4%)	327 (74.3%)	0.03*	0.0006*
3	255 (33.1%)	54 (46.6%)	113 (25.7%)		
Hepatitis B test	736 (95.6%)	113 (97.4%)	416 (94.5%)	0.50	0.30
OGTT 75 g	618 (80.3%)	98 (84.5%)	320 (72.7%)	0.003*	0.01*
Ultrasonography exam	737 (95.7%)	113 (97.4%)	410 (93.2%)	0.08	0.13

*ANCS* Antenatal care score, *TDAP immunization* tetanus, diphtheria, and acellular pertussis, *OGTT 75 g* oral glucose tolerance test; ITT: intention-to-treat, *PP* per protocol analysis

*p-values adjusted by multivariate analysis

^a^All IG (Intervention Group) includes all eligible pregnant women who received ANC in intervention primary health care units, regardless of the PRENACEL registration status;

^b^PRENACEL group only includes pregnant women who received and accessed PRENACEL SMS

^c^Reported as mean (standard deviation)

**Table 5:** Predictors of high antenatal care score - intention to treat analysis

Predictor	ANCS <42 points	ANCS ≥42 points	Crude RR (95% CI)	Adjusted RR (95% CI)^a^
Group - ITT				
Control PCHUs	75 (17.0%)	365 (83.0%)	1	1
Intervention PCHUs	91 (11.8%)	679 (88.2%)	1.05 (1.01–1.10)	1.05 (1.00–1.09)
Socioeconomic class				
Upper/Upper Middle	13 (11.3%)	102 (88.7%)	1	1
Middle	58 (10.0%)	523 (90.0%)	0.95 (0.89–1.02)	1.10 (0.97–1.25)
Lower	46 (16.3%)	236 (83.7%)	1.01 (0.95–1.08)	0.95 (0.88–1.01)
Illicit Drug use				
Yes	8 (25.8%)	23 (74.2%)	1	1
No	133 (12.4%)	935 (87.6%)	1.14 (1.01–1.29)	1.01 (0.95–1.08)

*ANCS* antenatal care score, *PHCU* Primary Health Care Units, *RR* Relative Risk, *ITT* Comparison of intervention group (women from intervention PHCUs, regardless of whether or not they had received any SMS) with control group

^a^RR also adjusted by the number of community health workers in each group

### Per-protocol analysis (PRENACEL group vs. control group)

Compared to the control group, the PRENACEL group included more women living with a partner (88.8% vs. 80%, *p* = 0.04), fewer women from higher social classes (5.6% vs. 14.7%, *p* = 0.02), and fewer women who reported illicit drug use (0% vs 4.3%, *p* = 0.02). There were no differences in other demographic characteristics between the PRENACEL and control groups (Table 3). Mean ANC score were higher in the PRENACEL group than in the control group [48.5 (4.2) vs. 45.2 (8.7), *p* < 0.0001]. Women in the PRENACEL group were also more likely to have ≥6 antenatal visits (96.6% vs. 84.8%, *p* = 0.001); to have folic acid (93.1% vs. 75.9%, *p* < 0.0001) and ferrous sulfate prescriptions (89.7% vs. 80%, p = 0.02); to get three serologic tests for syphilis (40.5% vs. 24.8%, *p* < 0.0001) and for HIV (46.6% vs. 25.7%, *p* < 0.0001); to get a 75 g OGTT (84.5% vs. 72.7%, *p* = 0.01); and to receive a dental consultation (57.8% vs. 40.5%, p = 0.01) (Table 4).

On univariate analysis, belonging to the PRENACEL group [RR = 1.14 (95%CI 1.06–1.22)], and not using illicit drugs [RR = 1.14 (95%CI 1.01–1.29)] predicted a high ANC score (i.e., ANC score ≥ 42). Conversely, not having a partner [RR = 0.93 (95%CI 0.89–0.98)], and having an unplanned pregnancy [RR = 0.93 (95%CI 0.89–0.98)] were negative predictors for a high ANC score (Table 6). On multivariate analysis (Table 6), only belonging to the PRENACEL group remained a positive predictor for a high ANC score [Adjusted per-protocol analysis, RR = 1.12 (95%CI 1.05–1.21)]. The PRENACEL NNT was seven.

**Table 6:** Predictors of high antenatal care score - per protocol analysis

Predictor	ANCS <42 points	ANCS ≥42 points	Crude RR (95% CI)	Adjusted RR (95% CI)
Group - PP				
Control	88 (20%)	352 (80%)	1	1
PRENACEL	7 (6%)	109 (94%)	1.14 (1.06–1.22)	1.12 (1.05–1.21)
Marital status				
Living with a partner	77 (16.3%)	394 (83.7%)	1	1
Not living with a partner	54 (71.1%)	22 (28.9%)	0.93 (0.89–0.98)	0.92 (0.85–1.00)
Socioeconomic class				
Upper/Upper Middle	9 (15.8%)	48 (84.2%)	1	1
Middle	27 (9.2%)	265 (90.8%)	0.95 (0.89–1.02)	0.96 (0.87–1.07)
Lower	18 (17.3%)	86 (82.7%)	1.01 (0.95–1.08)	1.06 (0.97–1.16)
Drug use				
Yes	2 (11.8%)	15 (88.2%)	1	1
No	66 (13.5%)	424 (86.5%)	1.14 (1.01–1.29)	0.98 (0.82–1.16)
Pregnancy intentions				
Planned pregnancy	23 (10.6%)	195 (89.4%)	1	1
Unplanned pregnancy	44 (15.8%)	234 (84.2%)	0.93 (0.89–0.96)	0.98 (0.92–1.04)

*ANCS* antenatal care score; Per protocol (PP) analysis: Compared PRENACEL group (only includes pregnant women who received and accessed PRENACEL SMS) with control group; *RR* Relative risk, *CI* Confidence interval

## Discussion

Our findings suggest that a bi-directional, mobile-phone based, short text message service is potentially useful to improve the coverage of recommended ANC practices, including syphilis and HIV testing. However, only one fifth of eligible women showed interest and registered in PRENACEL, suggesting that motivating pregnant women to enrol in such a program is one of the main obstacles that need to be tackled in order to achieve successful scale-up of this intervention.

Increasing coverage of recommended ANC practices maximizes the chance of identifying pregnancy complications, and of improving maternal and newborn outcomes [4, 27]. In Tanzania, Lund et al. [13] found that receiving text messages increased the number of women who had at least four antenatal visits by 13%. The increase in the percentage of women receiving more ANC visits is comparable to the 12.2% increase we found in the number of women having at least six antenatal visits with PRENACEL. In Kenya, SMS appointment reminders led women to attend a greater number of ANC visits, though the number of SMS required to make a difference was not specified [28]. In Brazil, 98.7% of pregnant women attend at least one ANC visit, and 75.8% attend six or more. In our study, a higher percentage of pregnant women in the control group attended more than six visits compared to the national average. It should be noted that this study was conducted in a Brazilian city with “Very High” Human Development Index (HDI) [29].

In the PRENACEL group, we noted a 15.7% increase in the number of pregnant women undergoing a third syphilis test compared to the control group. This is an important finding in light of recent increases in the incidence of congenital syphilis in Brazil (from 1.7 cases per 1000 live births in 2004 to 4.7 cases per 1000 live births in 2013) [30]. The Brazilian Health Ministry reported that more than 70% of children with congenital syphilis had mothers who had attended at least one antenatal visit; among those mothers diagnosed during antenatal visits, most received inadequate treatment [30]. Performing three syphilis tests during pregnancy creates opportunities for diagnosis and prevention of vertical transmission. However, in Brazil, syphilis testing coverage during pregnancy is 89.1% for one test and 41.2% for two. If a text messaging program such as PRENACEL can increase coverage of syphilis screening, its potential public health impact shouldn’t be neglected.

We also noted an increase in coverage of HIV testing, with PRENACEL accounting for a 15.4% and 20.9% increase, respectively, in the number of women undergoing two or three tests. Although recommended by Brazilian Health Ministry since 2006 [30], coverage of a second HIV screening test during pregnancy is still low (less than 30% at the national level but with marked regional differences) [7]. Testing rates for gestational diabetes were also increased in the PRENACEL group. To our knowledge, this is the first study examining the impact of an SMS messaging program on recommended ANC practices, including HIV, syphilis, and diabetes testing [31].

Our data show that even with a high proportion of women attending six or more antenatal visits, coverage was low for many of the recommended screening tests during pregnancy, suggesting that the quality of public ANC services is inadequate. Improving the qualification of health professionals and providing pregnant women with more information regarding the importance of these guidelines are strategies that could increase the effectiveness of ANC. Text messaging programs like PRENACEL could also be used to encourage women to participate in their own care and, for example demand tests that were not requested by her antenatal provider.However, our study also had some limitations. One is that the main data source used to evaluate the coverage of antenatal care practices was the ANC card, which could have been incomplete or improperly filled out by the health provider, thus failing to report all care received during the pregnancy [32]. The ANC card was nonetheless chosen because it is a national tool used for registering ANC exams and visits which allows for comparison with public health data [6–8]. We also anticipate that under-reporting of testing and visits on the card would occur to a comparable extent in both study groups, in view of the cluster randomization process. Another limitation is that despite performing a balanced randomization, there were differences between groups in variables not considered in the balancing process, such as the greater proportion of intervention PHCUs being in slum areas. However, we expect that this bias would underestimate, rather than overestimate, the effect of PRENACEL. The cluster randomization process and selection bias of women interested in receiving PRENACEL may have contributed to unbalanced individual characteristics. However, the favorable effects observed persisted after adjusting for unbalanced characteristics. Passive participant recruitment resulted in a low rate of voluntary registration in PRENACEL (20.4% of eligible women in intervention PHCUs). Although passive recruitment is a valid method of recruitment in pragmatic trials [33], the possibility of sampling bias should be considered, as women who voluntarily register in PRENACEL could also be prone to better engage in ANC.

In Brazil, 78.3% of the population owns a cell phone [34]. Given the potential impact in the coverage of ANC practices, further research is necessary to maximize the reach of similar mobile health programs.

## Conclusion

In conclusion, a bi-directional, mobile-phone based, short text message service is potentially useful to improve the coverage of recommended ANC practices, including syphilis and HIV testing. Further research focusing on how to maximize the reach of similar mobile health programs is recommended.

## Additional file

Additional file 1: Figure S1. Recruitment strategy. (PPTX 32 kb)

## Abbreviations

ANC: ANC
CI: Confidence interval
HDI: Human Development Index
ITT: Analysis intention-to-treat
MAMA: Mobile Alliance for Maternal Action
Mater: Women’s Health Reference Service Center
NNT: Number needed to treat
OGTT: Oral glucose tolerance test
PHCU: Primary health care units
PP: Analysis per-protocol
REBEC: Brazilian Clinical Trials Registry
REDCap: Record Electronic Data Capture
RR: Relative risks
SMS: Short message service
SUS: Brazilian Unified Health System
TDAP: tetanus-diphtheria-acellular pertussis
UNAERP: University of Ribeirão Preto
