# Supplementary material for: PRENACEL – a mHealth messaging system to complement antenatal care: a cluster randomized trial
Source: Reprod Health. 2017 Nov 7;14:146. doi: 10.1186/s12978-017-0407-1 (PMC5678588; doi:10.1186/s12978-017-0407-1)
Supplement: Additional file 1: Figure S1. — Recruitment strategy. (PPTX 32 kb) [file 12978_2017_407_MOESM1_ESM.pptx]

## Slide 1
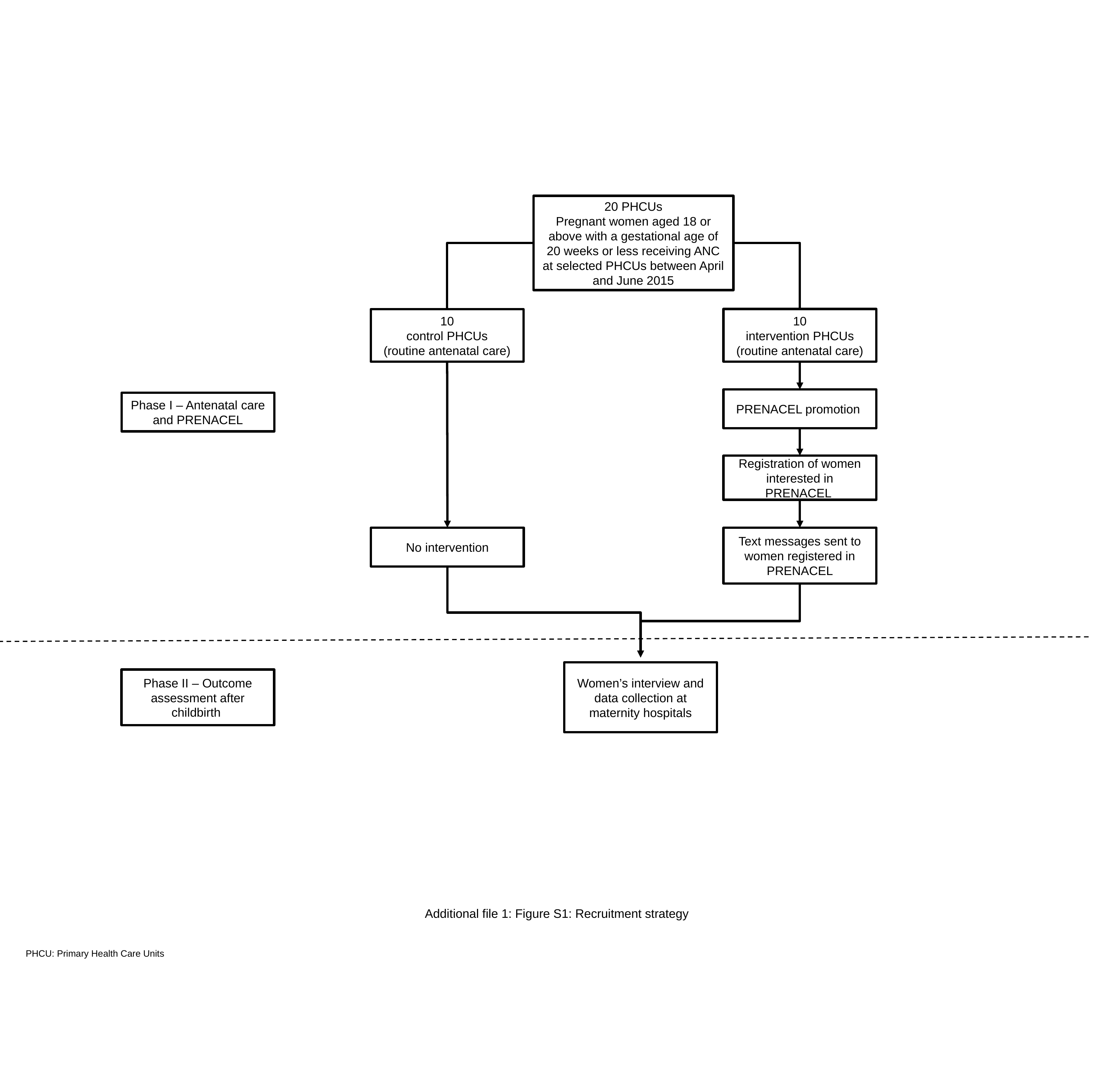

20 PHCUs
Pregnant women aged 18 or above with a gestational age of 20 weeks or less receiving ANC at selected PHCUs between April and June 2015
10
intervention PHCUs (routine antenatal care)
10
control PHCUs
(routine antenatal care)
PRENACEL promotion
Phase I – Antenatal care and PRENACEL
Registration of women interested in PRENACEL
No intervention
Text messages sent to women registered in PRENACEL
Women’s interview and data collection at maternity hospitals
Phase II – Outcome assessment after childbirth
Additional file 1: Figure S1: Recruitment strategy
PHCU: Primary Health Care Units
